# Methods Used to Assess Bull Sperm Chromatin Integrity and Its Correlation with In Vitro Embryo Production Efficiency

**DOI:** 10.3390/vetsci12020184

**Published:** 2025-02-18

**Authors:** Matheus Vicente Silva, Lays Oliveira Rocha, Bruno Augusto Nassif Travençolo, Ednaldo Carvalho Guimarães, Marcelo Emilio Beletti

**Affiliations:** 1 Faculdade de Medicina Veterinária, Universidade Federal de Uberlândia, Uberlândia 38400-902, Brazil; matheus.vicente@ufu.br; 2 Instituto de Ciências Biomédicas, Universidade Federal de Uberlândia, Uberlândia 38400-902, Brazil; lays.oliveira@ufu.br; 3 Faculdade de Computação, Universidade Federal de Uberlândia, Uberlândia 38400-902, Brazil; travencolo@ufu.br; 4 Instituto de Matemática e Estatística, Universidade Federal de Uberlândia, Uberlândia 38400-902, Brazil; ecg@ufu.br

**Keywords:** acridine orange, fertility, Feulgen reaction, toluidine blue, transmission electron microscopy

## Abstract

Sperm chromatin analysis is a crucial tool for investigating fertility in bulls, both in the field and in in vitro fertilization. This study aimed to identify efficient, low-cost, and easy-to-apply methods for detecting chromatin integrity alterations. Frozen semen samples from four bulls with varying results in in vitro embryo production (IVEP) were used. The sperm chromatin from these samples was evaluated using different methods: differences in the staining intensity with toluidine blue and the Feulgen reaction (FR), assessed both visually and computationally; visual evaluation of smears stained with acridine orange (AO) using epifluorescence and confocal microscopy; and evaluation using transmission electron microscopy (TEM) of sections positively stained with uranyl and lead. The same samples were utilized for IVEP. The results obtained using each method were correlated with one another and with the cleavage and embryonic development rates achieved in IVEPs. The odds ratio was employed to compare the chromatin alteration identification rates of the studied methods. Overall, chromatin integrity alterations identified using the AO and TEM methods exhibited correlations with the cleavage (r = −1.00) and embryonic development rates (r = −1.00), respectively. Among the methods tested, TEM and AO were the most effective for identifying chromatin integrity alterations that interfere with IVEP efficiency.

## Simple Summary

Sperm chromatin alterations are often a cause of subfertility in breeding bulls. Despite the significance of this pathology in bovine reproduction, it is frequently overlooked by veterinarians, as the standard method for evaluating chromatin alterations (SCSA—sperm chromatin structure assay) requires a flow cytometer, making it expensive and challenging to apply in field evaluations and assisted reproduction laboratories. This study tested alternative methods based on their efficiency, complexity, and cost, providing veterinarians with a basis for selecting the most appropriate method in each situation. The methods were as follows: difference in staining intensity with toluidine blue (TB) and the Feulgen reaction (FR), assessed both visually and computationally; visual evaluation of smears stained with acridine orange (AO) using epifluorescence and confocal microscopy; and evaluation using transmission electron microscopy (TEM). Among all the methods tested, AO staining and TEM were the most effective for identifying changes in chromatin integrity that interfere with in vitro production efficiency. The results of this study may reinforce the importance of employing techniques to identify chromatin alterations in bull sperm chromatin for selecting fertile animals, as sperm chromatin alterations in bulls are underexplored in breeding programs.

## 1. Introduction

During spermiogenesis, structural reorganization of chromatin occurs due to the replacement of histones by protamines, forming highly compacted chromatin capable of protecting deoxyribonucleic acid (DNA) integrity during sperm retention in the male and female reproductive tracts while silencing gene expression in protamine regions [1]. Changes in the structure and stability of sperm chromatin can lead to DNA damage, abnormal chromatin decompaction after fertilization, early embryonic development issues, and offspring health issues throughout life [2,3]. The impact of sperm chromatin changes on embryonic development depends on the severity of chromatin damage and the oocyte’s ability to repair DNA damage post-fertilization [4]. Therefore, understanding chromatin integrity is fundamental due to its influence on sperm fertilizing capacity and embryonic development. Efficient methods for analyzing sperm chromatin are necessary to maximize the effectiveness of male selection for natural reproduction and, particularly, for in vitro fertilization (IVF), aiming for greater efficiency in in vitro embryo production (IVEP). Traditional bovine semen analysis is not capable of assessing chromatin integrity, but specific methods exist for evaluating sperm chromatin. Sperm chromatin structure assays (SCSAs) are the most commonly used method for assessing chromatin integrity [5]. However, the influence of the changes identified using SCSA on IVEP efficiency is controversial. Some studies indicate no relationship between SCSA results, fertilization or pregnancy rates, and embryo quality [6,7]. Other studies demonstrate a relationship between SCSA results and cleavage and blastocyst rates [8]. Additionally, SCSA requires a flow cytometer, which limits its use in many cases and increases costs. Several alternative methods utilize dyes capable of detecting sperm chromatin condensation quality (toluidine blue (TB), acridine orange (AO), and Feulgen reaction (FR)) that do not require flow cytometry.

The Feulgen reaction was the first method used to evaluate bull sperm chromatin [9]. This method typically involves visual evaluation of semen smears, where sperm with altered chromatin compaction are more intensely stained, making the method highly subjective. Staining semen smears with TB after acid hydrolysis is another simple and low-cost method. This method has been shown to be good in identifying bulls with fertility problems [10]. It has also been tested in IVEP, revealing a correlation with blastocyst rate but not with cleavage rate (fertilization rate) [11].

Staining semen smears with TB after acid hydrolysis is another simple and low-cost method [12]. Like the FR, TB staining generally relies on visual assessment of subtle color differences, which introduces it with considerable subjectivity. Despite this subjectivity, it has yielded good results for evaluating bull fertility in the field [13]. By employing algorithms for image analysis of TB-stained smears, Hiraiwa et al. [14] reduced the subjectivity of the method and proposed a classification of sperm chromatin alterations based on their location in the sperm head. They found that only the most intense alterations interfere with IVEP [14].

Acridine orange, the fluorescent marker used in SCSA, can also be applied to semen smears to assess sperm chromatin. AO staining utilizes the metachromatic properties of the molecule to evaluate sperm chromatin stability through the susceptibility of sperm DNA to acid or heat denaturation. After acid hydrolysis or heat treatment, sperm with highly compacted chromatin remain and emit green fluorescence, while sperm with chromatin integrity alterations are susceptible to DNA denaturation, emitting red fluorescence. This method provides good results for identifying fertility problems in bulls in the field [7,15,16]. However, it has not demonstrated good results in identifying bulls with low efficiency in PIVE [7].

In addition to using dyes to evaluate sperm chromatin using light microscopy, transmission electron microscopy (TEM) can also be employed, as it is the ideal technique for studying sperm ultrastructure and characterizing ultrastructural defects that may influence fertilization and embryonic development [16,17]. Although it is a very expensive and labor-intensive technique, it has proven efficient in identifying alterations in sperm chromatin that affect fertility in humans [18]. Typically, TEM is used to identify alterations caused by external agents without testing the actual influence of these alterations on fertility in the field or in IVF [19]. Recently, Blandon et al. [20] used TEM to classify chromatin alterations in bull sperm affected by soluble antigens of Toxoplasma gondii, demonstrating that the most intense alterations significantly influence embryonic development in IVEP.

Considering the controversial or nonexistent results on the relationship between chromatin alterations identified using these different methods and IVEP efficiency, the present study aimed to find alternative methods to SCSA that are effective in identifying chromatin integrity that interferes with sperm fertilization capacity and early embryonic development in IVEP.

## 2. Materials and Methods

Frozen semen samples were used in TRIS-yolk medium from four Girolandos bulls (Bos taurus) with varying fertility grades. These samples, obtained from pasture-raised bulls with initially unknown fertility, were obtained using an artificial vagina and frozen in TRIS-yolk medium automatically in the TK equipment (Uberaba, MG, Brazil) model 3000 Compact, following the P2S1 freezing curve. The straws, each containing 500 µL with 40,000,000 motile sperms, were stored at −196 °C in liquid nitrogen.

Samples from a single batch of semen from each bull were used to perform routine IVEP and to evaluate chromatin compaction using all the methodologies tested in this study.

### 2.1. In Vitro Embryo Production (IVEP)

All semen samples were utilized for IVEP. Ovaries were collected from postmortem cows at a commercial slaughterhouse in Uberlândia. The IVEP production protocol was based on Hiraiwa et al. [14]. Oocytes (*n* = 1265) collected from ovaries using follicular aspiration underwent maturation, fertilization, and in vitro culture. Briefly, cumulus–oocyte complexes (COCs) were aspirated from follicles measuring 3 and 8 mm in diameter. COCs were recovered and morphologically classified according to cytoplasmic homogeneity and staining, number of layers, and cumulus cell appearance as viable (quality 1 and 2) or nonviable (quality 3 and 4) [21]. Only oocytes classified as grades 1 and 2 were used in the IVEP system.

The oocytes were subjected to in vitro maturation in modified TCM medium 199 (0.2 mM sodium pyruvate, 26 mM sodium bicarbonate, 83 µg/mL amikacin, 1 µg/mL FSH, 5 µg/mL LH) supplemented with 10% fetal calf serum (*v*/*v*). On average, 20 selected oocytes matured in drops (100 µL) over 22 h at 38.5 °C in an atmosphere of 5% CO_2_ and moisture saturated.

After maturation, the oocytes were washed three times in Fert-TALP medium (supplemented with penicillamine (21.1 mM), hypotaurine (10.4 mM), epinephrine (1 mM), heparin (10 mg/mL), and bovine serum albumin (BSA) (6 mg/mL)) and transferred to 100 µL drops. The drops were randomly distributed to compose the experimental groups, including the experimental bulls and the IVEP reference bull. For IVF, the sperm selection aimed to isolate viable sperm. A dose of semen was thawed in a water bath at 37 °C for 30 s and deposited on a Percoll gradient, previously prepared in a Falcon tube of 15 mL, consisting of a top layer of 45% Percoll (500 µL of 90% Percoll and 500 µL of TALP-Sperm medium (Tyrode–albumin–lactate–pyruvate, plus 0.2 mM sodium pyruvate and 83 µg/mL amikacin)) and a bottom layer of 1 mL of 90% Percoll (Percoll, 10× solution, 2 mM CaCl_2_ stock solution, 0.4 mM MgCl_2_ stock solution, 0.1 mM DL-lactic acid, and 20 mM sodium bicarbonate). The mixture was centrifuged for 30 min at 900× *g*. From the sediment resulting from the Percoll gradient selection, two 5 µL aliquots were taken to determine progressive motility and concentration in a Neubauer chamber. The concentration was adjusted to 25,000 viable sperms/µL, then 8 µL was used to fertilize the oocytes present in each drop. The samples were incubated for 18 to 20 h in an atmosphere of 5% CO_2_, 100% humidity, and a temperature of 38.5 °C. After IVF, the probable zygotes were mechanically denuded via successive pipetting and repeated washing in drops of 100 µL of synthetic oviduct fluid (SOF) medium (plus 0.2 mM sodium pyruvate, 5 mg/mL BSA, 2.5% fetal bovine serum, and 83 µg/mL amikacin sulfate) and transferred to 100 µL drops of the same medium, where they were cultivated for 6 days in an incubator at 38.5 °C in an atmosphere of 5% CO_2_ and moisture saturated.

The cleavage rate was determined 48 h after IVF by counting the zygotes that had two or more cells using a stereoscopic microscope. After the seventh day of fertilization, the embryonic development rate was assessed by counting the number of embryos that reached this stage of development relative to the number of cleaved oocytes. The blastocyst rate was also calculated, represented by the number of oocytes that were placed for maturation and the number of blastocysts obtained after 7 days of IVF. The data obtained using tested semen were normalized using the results from a fertile bull previously tested in our laboratories and with very stable results. This normalization aimed to neutralize IVEP variables that are not controllable. In all plates where IVEP routines were performed using the experimental semen samples, IVEPs were also conducted using semen from this reference bull (510 oocytes). We used normalization to transform the cleavage and embryonic development rates of the tested samples into percentages of the rates obtained with the reference bull’s semen in the same IVEP routine. Through the IVEP routines, it was verified that the four samples exhibited different performances in blastocyst production.

### 2.2. Chromatin Analysis

The sperm chromatin of the semen samples was evaluated using various methods: difference in staining intensity with TB and FR, assessed both visually and computationally, visual evaluation of smears stained with AO using epifluorescence and confocal microscopy, and evaluation using TEM.

#### 2.2.1. Smear Preparation

Several semen smears were prepared for analyses with TB, FR, and AO. For sperm smear preparation, one straw per bull was used to create smears after thawing in water at 37 °C for 30 s and selection based on Percoll gradient, as only samples that underwent Percoll selection were used in the IVF process. This approach ensured that only the spermatozoa participating in the IVF process were evaluated, allowing for an understanding of how chromatin changes affect sperm fertilizing capacity and early embryonic development. For Percoll gradient selection (45%/90%), the pellet was washed in TALP-sperm via centrifugation at 1300× *g* for 5 min. After the final wash, the pellet containing sperm was suspended in a volume of up to 150 µL, followed by homogenization. A volume of 10 µL was then pipetted to create the smear, and 3 smears were prepared using microscopic techniques, totaling 9 smears per bull. After smear preparation, fixation was performed by immersing them in a solution containing ethanol–acetic acid in a 3:1 ratio (Carnoy’s fixative) for 1 min, followed by washing the smear in 70% ethanol for 3 min [14].

#### 2.2.2. Visual Analysis and Image Capture from Toluidine Blue Staining

For the TB staining technique, after fixation, the smears were processed according to Hiraiwa et al. [14]. Using an Olympus EX51 microscope (Olympus, Tokyo, Japan) with an Olympus DP70 (Olympus, Tokyo, Japan) camera, employing a ×100 immersion objective and a ×2 optovar (intermediate lens), approximately 200 images of each smear were captured. Visual (subjective) analysis of normal cells (light blue to green) and chromatin decompaction (dark blue to magenta) was also performed, accounting for a total of 200 cells per smear.

#### 2.2.3. Visual Analysis and Image Capture from Feulgen Reaction Staining

For the FR staining technique, after fixation, acid hydrolysis was conducted in 4 N HCl at room temperature for 45 min. Following acid hydrolysis, the slides were washed in water and stained with Schiff’s reagent for 60 min at room temperature. Subsequently, solution baths of increasing concentrations of alcohol and xylene were applied. The Blade assembly was completed with Entellan. Using an Olympus EX51 microscope (Olympus, Tokyo, Japan) with an Olympus DP70 camera (Olympus, Tokyo, Japan) employing a ×100 immersion objective and a 2× optovar (intermediate lens), approximately 200 images were captured for each smear. Visual analysis (subjective) of normal cells (stained more weakly) and those with chromatin decompaction (stained more intensely) were also performed, accounting for a total of 200 cells per smear.

#### 2.2.4. Computer Analysis of Images from Feulgen Reaction and Toluidine Blue Staining

Feulgen reaction and TB-stained smears were also evaluated through computer image analysis. For this purpose, images of sperm from each smear were captured using an Olympus EX51 microscope coupled to an Olympus DP70 camera connected to a microcomputer via the DP Controller capture system, using a ×100 immersion objective and a ×2 optovar (intermediate lens).

After capturing the images, the sperm heads were segmented using histogram-based thresholding, forming a binary image (mask) of each sperm head. This technique aims to create an image containing only the sperm head, eliminating elements present in the original image such as the sperm tail or artifacts resulting from the staining process (25). Images containing only the sperm head underwent a process of discarding pixels present on the perimeter of the head and smoothing its contour using the mathematical technique known as erosion operation [20].

The segmented heads were evaluated using a program developed and executed in the Scilab programming environment, where the average pixel value of each head was obtained. To reference the normal stain (coloration) of the sperm heads, the program selected the 10 lightest (most compacted) and most homogeneous heads among hundreds segmented from each smear. That is, the heads with the highest average pixel values (the lightest) and the lowest coefficients of variation of pixel value (most homogeneous) were selected automatically. It is important to note that computational evaluation can be used to detect differences in color intensity that are not perceptible to the human eye [14]. The regions of the heads formed by pixels with a value of 7% or more below the reference value of the sample were considered regions with chromatin decompaction for TB staining. For the FR, regions with a value of 4% or more below the reference value of the sample were considered. To reduce noise and improve the delimitation of the altered region, a Gaussian filter was applied (sigma = 2 and radius = 5). Finally, to delineate the areas defined as decompacted, white lines were used. The classifications of chromatin integrity alterations according to location were performed based on the methodology used by Hiraiwa et al. [14], including base decompaction (BD); basal half decompaction (BHD); central axis decompaction (CAD); base–apex decompaction (BAD); total decompaction (TD); dispersed decompaction (DD); normal (N). All classifications were performed by the same evaluator.

#### 2.2.5. Confocal Microscopy of AO Florescence Test

The fixed smears were also used to perform the AO fluorescence test protocol. Acid hydrolysis was conducted with 4 N HCl at room temperature for 5 min. The slides were then washed in distilled water and dried at room temperature. Staining was performed by applying AO solution (0.1 M citric acid, 0.2 M Na_2_HPO_4_, 0.001 M EDTA, 0.15 M NaCl, LA stock 6 μg/mL in distilled water, pH 6), depositing 3 to 5 mL of the solution on the slide surface and incubating for 5 min. The smears were then washed in distilled water. Slide assembly was performed with glycerol, and the coverslip was sealed with nail polish. The slides were evaluated on the same day using an inverted laser scanning confocal microscope, ZEISS—LSM 510 Meta, Axiovert 200 M (Zeiss, Jena, Germany) using Zen Lite software 3.4 (Zeiss, Jena, Germany) with a ×63 immersion objective, 488 nm laser, and emission filter for the green pseudocolor (499–563 nm) and the red pseudocolor (574–681 nm). Digital images of approximately 150 sperm heads per smear were captured. Sperm heads with chromatin stability alterations were identified via visual observation of greater intensity of the red pseudocolor, and the percentage of heads showing denatured DNA per bull was calculated.

#### 2.2.6. Epifluorescence Microscopy of AO Florescence Test

A visual (subjective) evaluation was also performed using fluorescence microscopy of the heads of sperm stained with AO, totaling 150 heads per smear, as described by Tejada et al. [15]. The number of sperm heads exhibiting yellow or orange fluorescence (denatured DNA, with red overlapping green) was counted. This evaluation was also conducted using a Zeiss Axiovert 200 M microscope (ZEISS, Jena, Germany), but with the mercury lamp (HbO) present in the microscope and a blue excitation filter.

#### 2.2.7. Transmission Electron Microscopy (TEM)

Analysis of sperm chromatin integrity alterations using TEM was also performed. For each semen sample, four straws were thawed, placed in a 2 mL microtube, fixed in a 2.5% glutaraldehyde solution in 0.1 M phosphate buffer (pH 7.4), and incubated for 2 h. Subsequently, the samples were washed in pH 7.4 phosphate buffer solution for 5 min at 70× *g*. After washing, 500 μL of 1% osmium tetroxide solution was added for 30 min. The samples were then washed at 70× *g* for 5 min, and the supernatant was discarded. Next, 3% agar was added at 50 °C and placed in a refrigerator (4 °C) until the agar solidified. The material embedded in agar was cut into 1 mm³ fragments. The fragments were dehydrated for 5 min in baths of a solution of increasing concentrations of alcohol and then for 10 min in baths of 100% alcohol. Following this, the samples were transferred to three baths of propylene oxide for 10 min each, followed by inclusion in Epon EMS resin (Electron Microscopy Sciences, Hatfield, PA, UK). The material fragments were placed in a silicon mold, and a new Epon resin solution was added to complete the mold, which was then placed in an oven at 60 °C for at least 72 h for curing (polymerization) of the Epon resin. The samples were then cut using a Reichert-Jung ultramicrotome (Leica Microsystems Inc., Buffalo Grove, IL, USA), and the sections were placed on copper grids and contrasted with uranyl acetate and lead citrate, as described by Blandon et al. [20]. The sections were analyzed and photographically documented using a HITACHI HT 7700 transmission electron microscope (Hitachi, Ltd., Tokyo, Japan), and digital images of the sperm heads were obtained.

For the evaluation of the sperm heads in each sample, a minimum of 100 heads per sample were counted, allowing for the calculation of the percentage of occurrences of sperm chromatin changes following the classification criteria of Blandon et al. [20]. Each sperm head in the captured images was classified into one of the following grades: G1—presence of up to three small clear spots in the chromatin; G2—presence of up to six small clear spots or a clearer region occupying up to ¼ of the head; G3—presence of several clear spots (more than six) or a clearer region occupying up to half of the head; G4—presence of a clearer nuclear area occupying more than half or large, totally clear regions.

### 2.3. Statistical Analysis

Statistical analyses were performed using the open-access R program with a reference significance level of 5%. A multiple comparisons test was conducted between proportions to verify significant differences in cleavage and embryonic development rates among the bulls.

To analyze the results obtained using the staining techniques with TB, FR, AO, and TEM, the results were transformed into percentages relative to the total number of heads counted via the technique. To identify significant differences between the chromatin integrity alterations identified using the different evaluation methods, the data were subjected to analysis of variance (Two-way ANOVA) after normality testing (Shapiro–Wilk) and homogeneity of variance (O’Neill–Matthews). The methods were compared using the Scott–Knott test at a 5% significance level.

To assess how the sperm chromatin defects identified using the techniques influenced the cleavage rate (fertilization process) and the embryonic development rate (early embryonic development), Pearson’s correlation test was employed between the results for each variable and the rates of cleavage and embryonic development rates. To verify whether the different methods identified similar or different changes in chromatin integrity, Pearson’s correlation test was utilized. A significant difference was considered when *p* ≤ 0.05. To verify whether the rate of sperm with chromatin decompaction in one method was statistically equal to that of the other methods, the odds ratio (OR) was used.

## 3. Results

### 3.1. In Vitro Embryo Production

The results for normalized cleavage and embryonic development rates obtained in the IVEPs are shown in Table 1, demonstrating the varying levels of in vitro fertility among the bulls used in the experiment. For normalization, the cleavage and embryonic development rate results obtained from the semen samples of the evaluated bulls were transformed into a percentage of the cleavage and embryonic development rates obtained for the semen in the same IVEP routine.

A multiple comparisons test was performed between the proportions of cleavage rates and embryonic development of the bulls used in the experiment (Table 2 and Table 3).

Table 2 indicates that the cleavage rate of bull 4 significantly differs from that of the reference bull (*p* = 0.00002). Table 3 shows a significant difference between the embryonic development rates of bulls 3 and 4 compared to the embryonic development rate of the reference bull (*p* = 0.00002, *p* = 0.000006).

### 3.2. Chromatin Analysis

**Figure 1:**
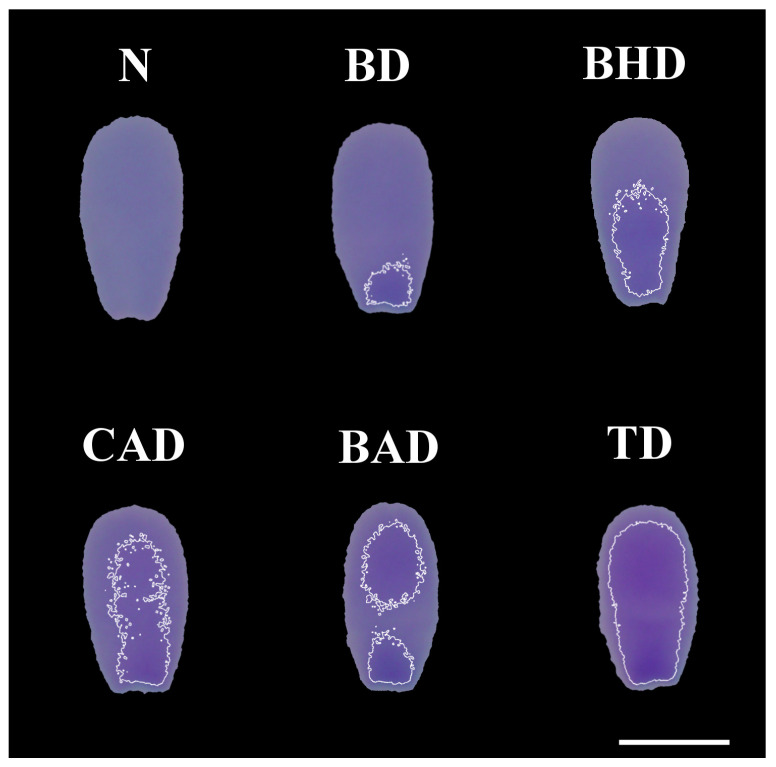
Different types of chromatin decompaction of sperm stained with TB. (N) normal; (BD) base decompaction; (BHD) basal half decompaction; (CAD) central axis decompaction; (BAD) base–apex decompaction; (TD) total decompaction. Scale bar: 5 μm.

Figure 1 presents examples of each type of sperm chromatin alteration identified via computational image analysis of the TB-stained smears.

**Figure 2:**
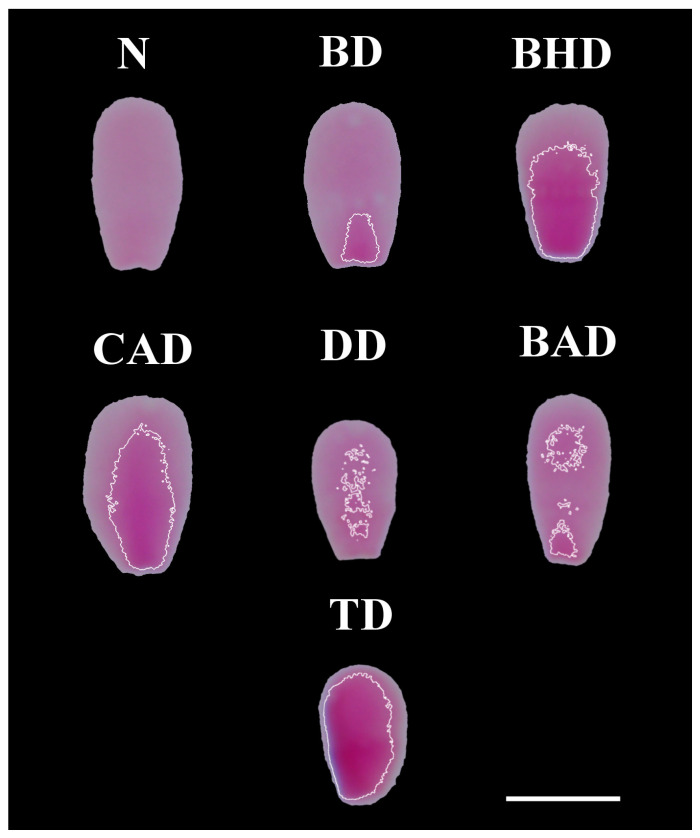
Different types of chromatin decompaction of sperm stained via the Feulgen reaction. (N) normal; (BD) base decompaction; (BHD) basal half decompaction; (CAD) central axis decompaction; (DD) dispersed decompaction; (BAD) base–apex decompaction; (TD) total decompaction. Scale bar: 5 μm.

Figure 2 shows examples of each type of sperm chromatin alteration identified through computational image analysis of the FR-stained smears.

**Figure 3:**
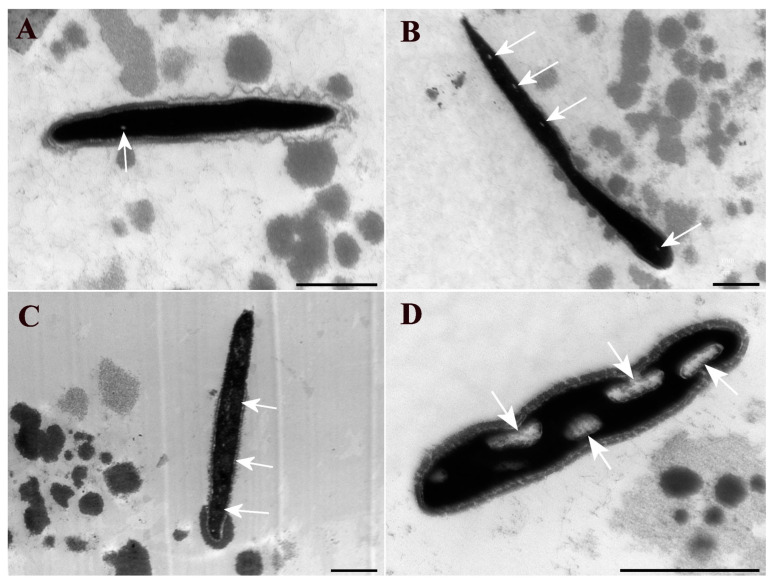
Electron micrographs of bovine sperm heads. (**A**) Observed grade chromatin integrity alteration is G1, characterized by the presence of up to three small clear spots in the chromatin; (**B**) grade chromatin integrity alterations G2, characterized by the presence of up to six small clear spots or a clearer region occupying up to ¼ of the head; (**C**) grade chromatin integrity alterations G3, characterized by the presence of several clear spots (more than six) or a clearer region occupying up to half of the head; (**D**) grade chromatin integrity alterations G4, characterized by the presence of a clearer nuclear area occupying more than half or large, totally clear regions. Areas with chromatin alterations are indicated by white arrows. Scale bar: 1 μm.

Figure 3 presents examples of each type of sperm chromatin alteration identified using TEM.

### 3.3. Correlation Between Chromatin Analysis Methods and IVEP

Table 4 shows that the AO sperm head staining results observed using laser scanning confocal microscopy (AOCM) and fluorescence microscopy (AOFM) demonstrated a significant negative correlation with the cleavage rate (r = −1.00, *p* = 0.004; r = −0.97, *p* = 0.03, respectively). Regarding TEM, a significant negative correlation was observed with the embryonic development rate (r = −1.00, *p* = 0.004).

### 3.4. Correlation Between Chromatin Analysis Methods

Pearson’s correlation test was employed to verify whether the chromatin analysis methods identified similar or different alterations in chromatin integrity (Table 5).

The computational evaluation of the smear stained with toluidine blue (CTB) showed a significant positive correlation with the visual evaluation of the smear stained with toluidine blue (VTB) (r = 0.98, *p* = 0.02), with the computational evaluation of the smear stained with FR (CFR) (r = 0.97, *p* = 0.03), and with the visual evaluation of the smear stained with FR (VFR) (r = 1.00, *p* = 0.003), as well as with the AO staining technique evaluated using fluorescence microscopy (AOFM) (r = 0.98, *p* = 0.02) (Table 5).

The VFR demonstrated a significant positive correlation with VTB (r = 0.99, *p* = 0.008), with CFR (r = 0.95, *p* = 0.04), and with AOFM (r = 0.97, *p* = 0.03). The AOFM exhibited a significant positive correlation with CFR (r = 0.95, *p* = 0.05) and with the AO staining technique evaluated using confocal microscopy (AOCM) (r = 0.99, *p* = 0.01). The AOCM showed a significant positive correlation with TEM (r = 0.95, *p* = 0.05) (Table 5).

### 3.5. Odds Ratio (OR)

Table 6 presents the OR for the results of the techniques used to assess chromatin integrity.

The CTB had 1.47 times more chance of identifying chromatin alterations compared to VTB (OR = 0.68, *p* = 0.0034). The CTB had a greater likelihood of identifying chromatin alterations compared to CFR and VFR (OR = 0.36, *p* = <0.0001; OR = 0.40, *p* = <0.0001, respectively). The TEM had 14.99 times more chance of identifying chromatin alterations compared to CTB (OR = 14.99, *p* = <0.0001) (Table 6).

It was observed that VTB had a greater likelihood of identifying chromatin alterations than the CFR and VFR techniques (OR = 0.53, *p* = <0.0001; OR = 0.59, *p* = 0.0016, respectively). In contrast, VTB had a lower chance of identifying alterations than AOCM, AOFM, and TEM (OR = 1.90, *p* = <0.0001; OR = 1.64, *p* = 0.0009; OR = 22.10, *p* = <0.0001, respectively) (Table 6).

The FR staining technique with computational evaluation had a lower chance of identifying chromatin alterations than AOFM, AOCM, and TEM (OR = 3.56, *p* = <0.0001; OR = 3.08, *p* = <0.0001; OR = 41.61, *p* = <0.0001, respectively) (Table 6). The VFR technique had a lower chance of identifying chromatin alterations than AOFM, AOCM, and TEM (OR = 3.21, *p* = <0.0001; OR = 2.78, *p* = <0.0001; OR = 37.53, *p* = <0.0001, respectively). The TEM had a higher chance of detecting chromatin alterations than AOCM and AOFM (OR = 11.69, *p* = <0.0001; OR = 13.50, *p* = <0.0001, respectively) (Table 6).

## 4. Discussion

The SCSA is considered the standard method for assessing changes in chromatin integrity [5], although the influence of changes identified via SCSA on IVEP efficiency is controversial [6,7,8]. Additionally, this requires a flow cytometer, which limits its use in many cases and increases the cost. There are cheaper alternative methods that are easy to apply. Some of these methods were tested in the present study.

In this study, we verified how the changes identified using the different methods specifically interfered with the fertilization capacity of sperm and early embryonic development. Computer analysis of the FR was utilized for the first time in this study to evaluate sperm chromatin. The other methods tested have been used in previous studies, but they were rarely or never found to be correlated with cleavage rates and embryonic development, which was accomplished in this article.

Among the methods tested, AO staining and TEM proved to be the most efficient techniques for identifying defects in sperm chromatin that interfere with sperm fertilization capacity and early embryonic development, as they exhibited the highest negative correlation coefficients with cleavage rates and embryonic development, respectively.

Several variations of TB staining were tested in this study. Although TB staining is the easiest and cheapest method to apply, it did not demonstrate high efficiency in identifying alterations in chromatin integrity that interfere with sperm fertilizing capacity or early embryonic development, as it did not show a significant correlation with cleavage rates and embryonic development. When comparing VTB with the CTB, a significant positive correlation was detected, which was expected because the visual evaluation does not involve classification.

The Feulgen reaction was the first method used to evaluate sperm chromatin associated with fertility problems in bulls [9]. However, this method has seldom been employed due to its subjective interpretation, as the difference between normal sperm and those with altered chromatin integrity is based on differences in staining intensity. Fernandes et al. [11] utilized visual evaluation of the FR and found that abnormal chromatin was associated with blastocyst rates in IVEP. In the present study, we tested the subjective visual assessment of Feulgen-stained smears and, for the first time, applied computational assessment algorithms originally used with TB to perform a more objective evaluation of Feulgen staining intensity. We also classified different types of chromatin decompaction based on the location of the decompacted areas. Performing the FR is a little more complex than staining with TB and incurs a similar cost; however, it did not prove to be highly efficient in identifying alterations in chromatin integrity that affect sperm fertilization capacity or early embryonic development, as it did not show a significant correlation with cleavage and embryonic development rates, either through computational analysis or visual assessment. Hiraiwa et al. [14] suggested, based on the observed correlation, that each classification type may exert different influences on bull fertility and IVEP.

When comparing the computational evaluations of TB and FR, we observe a significant positive correlation, which also held true for the visual evaluations between the two techniques. This demonstrates that both TB and FR can identify the same chromatin alterations. However, TB has a greater chances of detecting chromatin alterations (better performance) than FR, as observed through OR. A study by Beletti and Melo [22] found that visual evaluation of TB staining is a more efficient technique than FR for identifying chromatin alterations in rabbit sperm, as TB showed a greater capacity to distinguish between sperm with chromatin alterations (metachromasia) and normal sperm. In contrast, FR proved more susceptible to interpretation due to the variability in staining intensity observed in sperm.

To verify whether the alterations identified via AO and TB were similar, we performed a correlation analysis between these characteristics. The alterations identified using AOFM and CTB showed a significant positive correlation, indicating a high degree of similarity between the alterations identified using these techniques. This correlation was expected, as both techniques utilized acid hydrolysis-induced denaturation induced by acid hydrolysis, cationic dyes with metachromatic properties that bind to the phosphate groups of nucleic acids to assess sperm chromatin integrity [22,23]. However, the OR results indicated that AOFM and AOCM had a greater likelihood of identifying chromatin alterations (better performance) than VTB. These findings demonstrate that VTB is less efficient than AO and CTB, as it relies on a more subjective assessment and is unable to identify different types of chromatin decompaction of sperm stained with TB.

Correlations were also calculated between the variables evaluated with AO and FR. In this case, AOMF showed a significant positive correlation with VFR and CFR, demonstrating that there was great similarity between the alterations identified using these techniques. However, AO exhibited a greater likelihood of identifying chromatin alterations (better performance) than FR. The results of this study indicate that FR presented a lower sensitivity in identifying mild alterations compared to AO, even when employing computational evaluation of FR.

Acridine orange staining revealed significant negative correlations with the cleavage rate, indicating that chromatin alterations identified via AO staining mainly interfere with the ability to fertilize an oocyte. Transmission electron microscopy evaluations also showed significant negative correlations with the embryonic development rate, suggesting that chromatin alterations identified using TEM mainly affect early embryonic development.

Transmission electron microscopy is an ideal method for studying the ultrastructure of cells and various biological materials, and it is considered to be the best technique for examining the ultrastructure of spermatozoa and characterizing ultrastructural defects that may influence fertilization and embryonic development [17,20]. Through TEM, we were able to identify and classify chromatin changes in bovine sperm into five categories [20]. However, the use of TEM is limited in andrology laboratories because its methodology is difficult to implement, as it requires highly qualified technical support and expensive equipment and supplies [14]. Furthermore, our study found that TEM only allows for observation of sperm head sections with a thickness of 60 to 90 nm, which restricts the observed area. Consequently, a large number of evaluated heads are necessary to minimize this bias; in the present study, approximately 100 heads were evaluated per semen sample.

In this study, the AOMC and AOMF evaluations showed significant negative correlations with the cleavage rate. Therefore, changes in chromatin integrity identified via smears analyzed through visual assessment using fluorescence microscopy and confocal laser scanning microscopy characterize semen with low in vitro fertilization capacity. In both analyses, we were able to differentiate between green (intact DNA) and red (denatured DNA) spermatozoa with minimal subjectivity. This allowed us to obtain results that correlated with IVEP outcomes. Both evaluations exhibited a high negative correlation with the cleavage rate. Although some oocytes may initiate cleavage without fertilization, there is a strong relationship between the cleavage rate and the fertilizing capacity of spermatozoa [24]. Thus, the chromatin integrity alterations identified using these two methods characterize semen with low in vitro fertilization capacity. While there is not a direct and absolute relationship between bulls’ performance in the field and their IVEP efficiency, a correlation typically exists between IVEP success and bull fertility in the field [14], suggesting that these methods are likely effective in identifying bulls with fertility issues related to their sperm’s fertilizing capacity.

To determine whether AOCM, AOFM, and TEM techniques identify similar alterations, we performed a correlation analysis between these methods. However, a significant correlation was found only between TEM and AOMC. These findings indicate that the alterations identified using TEM are similar to those identified using AOMC. Nonetheless, TEM had a greater chance of identifying chromatin alterations (better performance) than AO methods, as reflected in the OR values.

The acridine orange and TEM techniques were more likely to identify chromatin alterations (better performance) than the TB and FR staining techniques. These results highlight the high capacity of TEM and AO to detect chromatin alterations, as these techniques exhibit greater precision and lower subjectivity. Undeniably, TEM is more accurate in identifying chromatin alterations than the other techniques, primarily due to its efficiency in detecting sperm ultrastructures that may influence fertilization potential [17,20] and its ability to identify and classify chromatin alterations in bovine sperm with greater precision [20].

The results of this study may reinforce the importance of using techniques to identify alterations in bull sperm chromatin for the selection of fertile animals, as chromatin alterations in bull sperm are often overlooked in breeding programs. Our findings may also contribute to future studies aimed at enhancing the efficiency and accessibility of TB, RF, AO, and TEM techniques for field use, as well as the development of new methods that are capable of identifying sperm chromatin alterations, thereby deepening our understanding of how identifying these alterations is a crucial tool for reproductive efficiency. Furthermore, this study opens the possibility of incorporating the AT, RF, AO, and TEM techniques in bull selection programs.

All correlation coefficients were negative and very high, with the smallest being −0.766 and the largest −0.966. However, only coefficients above 0.970 were considered significant (*p* ≤ 0.05) due to the limited number of samples tested. If a larger sample size had been evaluated, it is likely that all correlations would have reached significance. Additional studies are needed to explore potential correlations between the types of sperm chromatin alterations identified using the TB and the FR techniques, as well as AO with IVEP, and to determine any significant correlations between techniques that could not be utilized in this study due to the small number of semen samples.

## 5. Conclusions

The results of the present study indicate that, among the methods tested, the evaluation of chromatin alterations using TEM and AO staining assessed through fluorescence microscopy and confocal laser scanning microscopy were the most effective in identifying chromatin alterations that interfere with the efficiency of in vitro production of bovine embryos. However, TEM has limitations for routine laboratory use and field application, as it is very expensive, requires a lengthy sample processing time, and necessitates a qualified professional to perform the technique. In addition, the techniques utilizing smears stained with TB and FR are less effective than TEM and AO, they are more cost-effective, and they can be used with relative effectiveness to identify sperm chromatin alterations.

## Figures and Tables

**Table 1:** Cleavage, embryonic development, and blastocyst rates obtained from semen samples from bulls normalized with cleavage and embryonic development rates obtained from the semen from the reference (fertile) bull.

Bull	Cleavage Rate (%)	Normalized Cleavage Rate (%)	Embryonic Development Rate (%)	Normalized Embryonic Development Rate (%)	Blastocyst Rate	Normalized Blastocyst Rate
1 R. Bull	48.53 56.56	85.81	37.37 47.83	78.15	18.14 27.05	67.06
2 R. Bull	57.67 51.69	111.57	60.48 55.74	108.52	34.88 28.81	121.07
3 R. Bull	36.13 48.05	75.19	3.57 50.00	7.14	2.58 24.03	10.74
4 R. Bull	34.25 68.97	49.67	3.23 53.75	6.00	2.17 37.07	5.85

R. Bull: reference bull.

**Table 2:** Multiple comparisons test between bull cleavage rate proportions.

Bull	R. Bull	1	R. Bull	2	R. Bull	3	R. Bull	4
R. Bull	0.56557	1.96836	0.56737	0.03885	1.97009	11.39723	3.66248	14.50282
1	0.96157	0.48529	0.29968	3.50222	0.00800	5.41824	12.35536	7.81876
R. Bull	0.99916	0.99990	0.51695	1.08975	0.35470	6.49411	6.98008	8.69244
2	1.00000	0.83499	0.99325	0.57674	3.32374	16.72623	3.84293	21.56388
R. Bull	0.96148	1.00000	0.99982	0.85353	0.48052	4.39319	11.57690	6.33717
3	0.12221	0.60906	0.48338	0.01925 *	0.73354	0.36129	28.61902	0.11742
R. Bull	0.81773	0.08946	0.43096	0.79768	0.11536	0.00017 *	0.68966	34.07570
4	0.04293 *	0.34885	0.27550	0.00302 *	0.50098	1.00000	0.00002 *	0.34254

R. Bull: reference bull. The main diagonal displays the proportions (which, when multiplied by 100, become %). The upper part of the main diagonal contains the test statistics, while the lower part shows the *p*-values. * Significant difference when *p* < 0.05.

**Table 3:** Multiple comparisons test between proportions of the embryonic development rate of bulls.

Bull	R. Bull	1	R. Bull	2	R. Bull	3	R. Bull	4
R. Bull	0.478261	1.822630	0.831499	2.914235	0.069235	24.839380	0.533416	26.652649
1	0.969002	0.373737	5.222444	12.062930	2.769913	16.767950	4.868526	18.240028
R. Bull	0.997109	0.632838	0.557377	0.377887	0.451648	32.599230	0.056105	34.787848
2	0.892821	0.098508	0.999782	0.604839	2.089908	51.268120	0.904705	55.599319
R. Bull	0.999999	0.905440	0.999605	0.954697	0.500000	28.193090	0.221799	30.282577
3	0.000810 *	0.018955 *	0.000031 *	0.000000 *	0.000203 *	0.035714	34.030740	0.001442
R. Bull	0.999315	0.676004	1.000000	0.996223	0.999964	0.000017 *	0.537500	36.583717
4	0.000385 *	0.010933 *	0.000012 *	0.000000 *	0.000084 *	1.000000	0.000006 *	0.032258

R. Bull: reference bull. The main diagonal displays the proportions (which, when multiplied by 100, become %). The upper part of the main diagonal contains the test statistics, while the lower part shows the *p*-values. * Significant difference when *p* < 0.05.

**Table 4:** Pearson correlation coefficients between cleavage rate and embryonic development with chromatin alterations identified via computational and visual evaluation of smears stained with TB and FR, AO staining technique, and TEM.

Techniques	Cleavage Rate	Embryonic Development Rate
CTB	−0.909	−0.903
VTB	−0.853	−0.929
CFR	−0.859	−0.766
VFR	−0.881	−0.908
AOCM	−0.996 *	−0.919
AOFM	−0.971 *	−0.911
TEM	−0.922	−0.996 *

CTB, computational evaluation of TB; VTB, visual evaluation of TB; CFR, computational evaluation of FR; VFR, visual evaluation of FR; AOCM, AO evaluated using confocal microscopy; AOFM, AO evaluated using fluorescence microscopy; TEM, transmission electron microscopy. * Significant correlation when *p* ≤ 0.05.

**Table 5:** Pearson correlation between techniques used to assess bull sperm chromatin integrity.

	CTB	VTB	CFR	VFR	AOCM	AOFM	TEM
CTB	1.00	0.98 *	0.97 *	1.00 *	0.94	0.98 *	0.93
VTB	-	1.00	0.91	0.99 *	0.88	0.94	0.94
CFR	-	-	1.00	0.95 *	0.89	0.95 *	0.82
VFR	-	-	-	1.00	0.92	0.97 *	0.93
AOCM	-	-	-	-	1.00	0.99 *	0.95 *
AOFM	-	-	-	-	-	1.00	0.94
TEM	-	-	-	-	-	-	1.00

CTB, computational evaluation of TB; VTB, visual evaluation of TB; CFR, computational evaluation of FR; VFR, visual evaluation of FR; AOCM, AO evaluated using confocal microscopy; AOFM, AO evaluated using fluorescence microscopy; TEM, transmission electron microscopy. * Significant correlation when *p* ≤ 0.05.

**Table 6:** Odds ratio (OR) and 95% confidence intervals (95%CI) for the results of the techniques used to assess chromatin integrity.

Techniques		OR	95% CI	*p*-Value
	VTB	0.68	0.53–0.88	0.0034
	CFR	0.36	0.28–0.47	<0.0001
CTB	VFR	0.40	0.30–0.54	<0.0001
	AOCM	1.28	1.00–1.64	0.0548
	AOFM	1.11	0.86–1.43	0.4552
	TEM	14.99	11.67–19.24	<0.0001
	CFR	0.53	0.40–0.71	<0.0001
	VFR	0.59	0.43–0.81	0.0016
VTB	AOCM	1.90	1.43–2.50	<0.0001
	AOFM	1.64	1.23–2.18	0.0009
	TEM	22.10	16.63–29.36	<0.0001
	VFR	1.11	0.80–1.54	0.5946
CFR	AOCM	3.56	2.68–4.73	<0.0001
	AOFM	3.08	2.31–4.12	<0.0001
	TEM	41.61	31.19–55.52	<0.0001
	AOCM	3.21	2.34–4.41	<0.0001
VFR	AOFM	2.78	2.02–3.84	<0.0001
	TEM	37.53	27.26–51.68	<0.0001
AOCM	AOFM	0.87	0.66–1.14	0.3427
	TEM	11.69	8.87–15.39	<0.0001
AOFM	TEM	13.50	10.19–17.88	<0.0001

CTB, computational evaluation of TB; VTB, visual evaluation of TB; CFR, computational evaluation of FR; VFR, visual evaluation of FR; AOCM, AO evaluated using confocal microscopy; AOFM, AO evaluated using fluorescence microscopy; TEM, transmission electron microscopy.

## Data Availability

The raw data supporting the conclusions of this article will be made available by the authors on request.
